# The Diagnostic Accuracy of Radionuclide Arthrography for Prosthetic Loosening in Hip and Knee Arthroplasty

**DOI:** 10.1155/2013/693436

**Published:** 2013-07-15

**Authors:** Timothy H. French, Nicholas Russell, Anand Pillai

**Affiliations:** ^1^Department of Orthopaedic Surgery, Royal Adelaide Hospital, Australia; ^2^Department of Orthopaedics, University Hospital of South Manchester, M23 9LT, UK

## Abstract

*Purpose of Study*. Diagnosis of prosthetic loosening in hip and knee arthroplasty remains a challenge. Although there are a number of diagnostic tools, no single test or combination is 100% sensitive or specific. There has been a recent interest in the use of radionuclide arthrography (RNA) for detection of prosthetic loosening. *Methods*. A retrospective review of 45 consecutive RNA scans from 2005 to 2010 was conducted. RNA findings were compared with intraoperative findings at revision and/or serial radiographic examinations to confirm loosening. A component was considered loose if sequential radiographs demonstrated macromotion, gross subsidence, or progressive radiolucency. *Results*. There were 26 females and 17 males, with mean age at RNA of 71 years (range of 53–89 years) and mean time from index surgery, 6.4 years (range of 0.5–23 years). There were 23 total knee replacements (TKR) (19 primary and 4 revision) and 20 total hip replacements (THR) (11 primary and 9 revision). 15 patients underwent revision surgery following RNA. Strict inclusion criteria allowed 27 patients for further analysis. Sixteen RNA scans were suggestive of loosening, of which 14 were confirmed loose. Eleven scans were suggestive of a stable prosthesis, of which 10 were confirmed well fixed. RNA had a sensitivity of 93%, specificity of 83%, positive predictive value of 88%, and negative predictive value of 91%. *Conclusion*. Radionuclide arthrography should be considered a useful adjunct in the diagnosis of prosthetic loosening in the challenging patient.

## 1. Background

The incidence of prosthetic loosening is increasing with the growing number of hip and knee arthroplasty [1–3]. In Australia, there were 34,108 primary TKR's in 2009, an increase of 4.3% from 2008. The cumulative revision at 9 years for primary conventional TKR (for OA) is 5.1%, with loosening/lysis of the implant being the most common reason for revision. Likewise for primary THR, the total number in 2009 was 23,682, an increase of 4.2% from 2008. The cumulative revision at 9 years for primary conventional THR (for OA) is 5.2%, again with loosing/lysis being the most common reason [4].

The diagnosis of prosthetic loosening remains a challenge for the treating clinician, particularly differentiating between septic and aseptic prosthesis loosening [1, 5–8]. At our institution, routine diagnostic workup of a patient with suspected prosthetic loosening includes a thorough clinical examination, serum laboratory testing, and imaging modalities including plain radiography, computer tomography, and bony scintigraphy. When the diagnosis remains in doubt after routine workup, RNA has been utilised. The literature on RNA is scant, with few recent larger studies being conducted on its value. Several smaller studies have however commented on its potential benefit and emerging application in the clinical setting [9–11]. The aim of this study is to determine the overall diagnostic accuracy of RNA in both hip and knee prosthetic loosening.

## 2. Method

A retrospective review at the Queen Elizabeth Hospital in Adelaide, south Australia, identified 45 consecutive RNA scans performed from January 2005 to April 2010, which encompassed all RNA scans during this period. These scans were performed to investigate suspected loosening of knee or hip arthroplasty. In order to confirm the validity of the RNA result, the following inclusion criteria were required: revision surgery within 1 year of the RNA, *OR*, serial radiological follow-up (an index X-ray of the implant at the time of RNA and at least 2 further radiographs at 6 m and 12 m after RNA). Patients were also required to have either a TKR or THR.

Sixteen patients were excluded due to insufficient radiological follow-up, where no serial radiographs could be identified. A further 2 patients were excluded with hemi-arthroplasties.

This left a total of 27 patients for analysis. Twelve RNA scans were compared to revision findings at surgery (44%), and 15 were compared to radiograph series (56%). 

RNA technique was carried out under fluoroscopic guidance and aseptic conditions. An 18-gauge needle was inserted laterally between the femoral condyle and the patella into the knee joint space and a lateral approach to the hip joint. A small amount of contrast was injected if necessary to confirm appropriate positioning before the colloid injection [11]. 50 MBq in 2-3 mL of Tc-99c calcium phytate colloid (Radpharm, Australia) was injected through the needle before withdrawal. 

**Figure 1:**
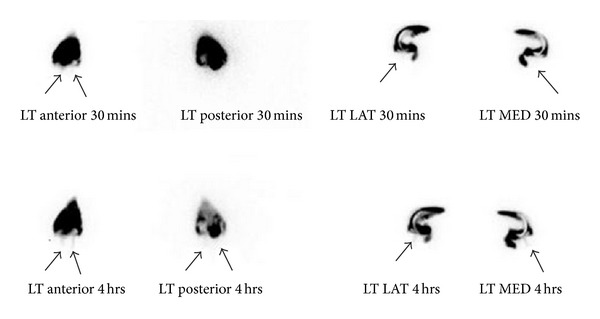
Example of left knee RNA, tibial component loosening with tracer leakage (arrows).

Anterior, posterior, medial, and lateral images of the knees and anterior, posterior, lateral, anterior oblique, and posterior oblique images of the hips were obtained approximately 30 min and 4 h after injection. The patient was encouraged to ambulate between imaging times. Further images were obtained at 24 h if necessary to obtain a good tracer image [11]. Loosening was considered to be present with tracer leakage around the prosthesis and a change in appearance over time (see Figure 1).

### 2.1. Analysis

Each RNA scan was classified into “positive” or “negative” for loosening. Classification was determined based on interpretation by a nuclear medicine specialist and the subsequent RNA report. 

Each patients' radiograph series or revision findings were classified into “loosening confirmed” or “arthroplasty stable.” Loosening on radiograph series was confirmed separately by 2 orthopaedic fellows. A component was considered loose if sequential radiographs demonstrated macromotion, gross subsidence, or progressive radiolucency of >2 mm at interfaces. Classification described by Harris et al. was utilized for femoral component loosening [12]. Loosening confirmed via revision surgery was determined by operation notes and was based on the subjective effort required to extract implants. Classification of findings was blinded from the previous results of the RNA. This study was performed with the approval of the Queen Elizabeth Hospital Ethics of Human Research Committee.

## 3. Results

A total of 27 patients were included in the analysis. There were 16 females and 11 males, with a mean age of 71 years. The mean time from index surgery to RNA was 6.5 years, with a range of 6 months to 23 years. There were 13 TKR and 14 total THR. Of the 13 TKR, 11 were primary replacements and 2 were revisions, 3 were cemented, 5 were uncemented and 5 were hybridised. Of the 14 THR, 8 were primary replacements and 6 were revisions, 4 were cemented, 3 were uncemented, and 7 were hybridised with a cemented stem and uncemented cup.

Twelve RNA scans were compared to revision findings at surgery (44%), and 15 were compared to radiograph series (56%). 

**Figure 2:**
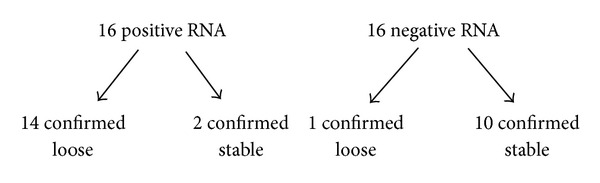
Schematic of results.

There were 16 positive RNA results. Of these, 14 were confirmed loose by radiograph series or revision findings, and 2 were confirmed stable. There were 11 negative RNA results. Of these, 10 were confirmed stable and 1 was confirmed loose (see Figure 2).

This gave a true positive of 14 out of 16 results and a true negative of 10 out of 11 results. The calculated sensitivity of RNA in our study was 93%. The calculated specificity was 83%. Sensitivity was calculated as True positives/(True positives + False negatives). Specificity was calculated as True negatives/(True negatives + False negatives).

The positive predictive value (PPV) and negative predictive value (NPV) are 88% and 91%, respectively.

## 4. Discussion

There are a number of tools available to the orthopaedic surgeon for the diagnosis of prosthesis loosening; however, no single tool or combination of tools has been found to be 100% sensitive or specific [13]. Available imaging used in combination with clinical examination and laboratory tests includes plain radiography, subtraction arthrography, nuclear arthrography, bone scintigraphy, and recently single-photon emission tomography (SPECT) and positron emission tomography (PET) with F-fluorodeoxyglucose (FDG) [2, 5]. However controversy still exists about the relative utility of these investigations [2].

In recent years, RNA has been used at our institution as an adjunct in the diagnosis of prosthetic loosening. The aim of this study was to determine if it added value to the standard workup of such patients. In 2006, Temmerman et al. reported a sensitivity of 69% and a specificity of 76% for nuclear arthrography and concluded that, when used in conjunction with plain radiography, it made a significant contribution to the diagnosis [2]. Kitchener et al. reported overall sensitivity of 88% and a specificity of 88% of RNA for the diagnosis of TKR loosening, with a gold standard of patients who underwent surgical correlation or arthroscopic assessment [11]. A similar study assessing RNA in hip arthroplasties was conducted by Miniaci et al. which showed good sensitivity and specificity (85.7% and 87.5%, resp.) of RNA in diagnosing femoral component loosening but poorer values for diagnosing acetabular component loosening (sensitivity of 48.3% and specificity of 66.7%) [10]. Several studies have similarly questioned RNA in diagnosing acetabular component loosening; however, a meta-analysis of 32 studies conducted in 2005 by Temmerman et al. showed a comparable sensitivity and specificity of RNA for acetabular loosening of 85% and 83%, respectively [14, 15]. Other studies have demonstrated variable sensitivities and specificities of RNA in both hip and knee prosthetic loosening [2]. The present study demonstrates similar results to those previously published, with a sensitivity of 93% and a specificity of 83% in a selected group of patients.

Limitations of this study include the limited sample size and the selection of patients for which RNA is typically used in our institution. Our limited sample size will alter the accuracy of sensitivity, specificity, PPV, and NPV, and larger studies are needed to provide support to these results. In addition, patients selected for RNA are typically those who have a history suggestive of loosening, with clinical examination, plain radiograph, and whole body bone scan (WBBS) unable to provide a definitive diagnosis. Unfortunately this means that no RNA research had yet been produced as a first-line detection modality on a randomised group of patients with TKR or THR.

Another limitation to the study design is the use of plain radiograph series or revision operative findings as a gold standard to compare with the accuracy of RNA. The sensitivity and specificity of serial radiographs are limited, with some reports suggesting varied sensitivities of between 85% and 95% [16–18]. In some instances, the diagnosis of loosening may be difficult even at the time of revision surgery. Currently, there exist no defined criteria for prosthetic loosening at the time of revision surgery.

A known concern of RNA as a diagnostic tool is the potential to introduce infection into the joint space. At our institution, however, there has not been a single reported incidence of this complication from the 45 RNA studies surveyed between 2005 and 2010. RNA requires specialised training in correct administration technique and analysis of images retrieved as it is not a mainstream modality. Currently, there is a lack of standard criteria for interpretation of RNA, and hence this may result in poor reproducibility of RNA results. This study highlights the need for the development of diagnostic criteria, which will allow the wider use of RNA and more consistent interpretation of results.

A potential advantage that RNA may offer is the option of concurrent synovial aspiration in cases with a potential infectious aetiology. We recommend this as a routine additional diagnostic tool. Future potential for RNA has also been mentioned in combination with the newer SPECT or SPECT/CT technology, which allows for 3D images to be taken of the joint and is used concurrently with identification of RNA tracer leakage. In a recently published article, Chew et al. discuss the emerging applications of this technology [19].

There may be additional cost benefits associated with RNA in the decision to delay or not proceed with revision surgery if a finding negative of loosening is reported. However, no cost versus benefit data is available at this time and is highly institution specific.

## 5. Conclusion

We conclude that RNA should be considered as an additional diagnostic tool for use in difficult cases of suspected prosthetic loosening and that it is a safe and effective diagnostic modality with high accuracy. Further studies with larger numbers of patients and follow-up will be required to define a standardised criterion for its interpretation.
